# Thermal performance curves and age-stage, two-sex life table of *Dermatophagoides farinae* (Acari: Pyroglyphidae): baseline data for climate change impact assessment

**DOI:** 10.3389/finsc.2026.1913734

**Published:** 2026-08-19

**Authors:** Zhimin Xu, Xu Zhang, Zhibin Lin, Huiquan Lin, Yongwen Lin, Haiming Cai

**Affiliations:** 1 Pediatric Department, Zhangzhou Affiliated Hospital of Fujian Medical University, Zhangzhou, China; 2 College of Food Engineering, Zhangzhou Institute of Technology, Zhangzhou, China

**Keywords:** climate change, *Dermatophagoides farinae*, house dust mites, intrinsic rate of increase, life table, temperature

## Abstract

**Introduction:**

House dust mites (HDMs) are major indoor allergens worldwide, with *Dermatophagoides farinae* being one of the most prevalent species. Temperature is a key driver of mite development, but the thermal biology of HDMs remains poorly characterized. Despite the medical importance of house dust mites, comprehensive thermal performance data across ecologically relevant temperature ranges remain limited, particularly for upper thermal thresholds that will become increasingly relevant under climate warming.

**Methods:**

We evaluated the development, survival, and fecundity of *D. farinae* under five constant temperatures (15, 20, 25, 30, and 35°C) at 75 ± 5% relative humidity using the age-stage, two-sex life table framework.

**Results:**

Developmental duration decreased significantly from 15 to 30°C but increased at 35°C due to thermal inhibition. The shortest pre-adult development time was 19.14 days at 30°C, while the longest was 68.98 days at 15°C. Egg-to-adult survival was highest at 25°C (88.6%) and lowest at 35°C (35.7%). The net reproductive rate (R_0_) peaked at 25°C (33.96 offspring per female), whereas the intrinsic rate of increase (r_m_) was highest at 30°C (0.0598 day^−1^), reflecting the differential contributions of fecundity versus developmental speed to population performance. *D. farinae* exhibits optimal development at 25–30°C, with severe thermal stress above 35°C.

**Discussion:**

These baseline thermal performance data provide essential parameters for population models aimed at assessing climate change impacts. Our results suggest that climate warming may initially increase HDM populations in temperate regions but could restrict their distribution in areas where thermal thresholds are exceeded. These findings provide baseline data for assessing allergen exposure risks under future climate scenarios.

## Introduction

House dust mites (Acari: Pyroglyphidae) are cosmopolitan arthropods that proliferate in human-built environments (1). Among the approximately 20 known pyroglyphid species associated with human dwellings, *Dermatophagoides farinae* (Hughes, 1961) (American house dust mite) and *D. pteronyssinus* (Trouessart, 1897) (European house dust mite) are of the greatest medical relevance due to their production of potent allergens (2, 3). *D. farinae* primarily inhabits mattresses, pillows, carpets, upholstered furniture, and stuffed toys, where it feeds on shed human skin scales, fungal spores, and organic detritus. Globally, *D. farinae* has a cosmopolitan distribution with higher prevalence in temperate and subtropical regions, and it tends to dominate in drier indoor microenvironments compared to *D. pteronyssinus*. These allergens are major etiological agents of allergic diseases including asthma, allergic rhinitis, and atopic dermatitis, affecting an estimated 10–20% of the global population (4–6). Major allergen groups include Group 1 (Der f 1, a cysteine protease found in fecal pellets) and Group 2 (Der f 2, a lipid-binding protein from mite bodies), along with additional groups (Der f 3–23) comprising proteases, structural proteins, and metabolic enzymes (7–9). Sensitization to HDM allergens is strongly associated with allergic asthma, perennial allergic rhinitis, atopic dermatitis, and allergic conjunctivitis, and early-life exposure is a recognized risk factor for the development of allergic sensitization and asthma. Common preventive measures include regular hot-water washing of bedding, use of allergen-impermeable mattress and pillow encasements, reduction of indoor relative humidity below 50%, and frequent vacuuming with HEPA filters (10, 11).

Temperature and relative humidity are the primary abiotic factors shaping the distribution, abundance, and seasonal dynamics of house dust mites (6, 12). As poikilothermic organisms, mites rely entirely on environmental heat to fuel metabolic processes, growth, and reproduction. Thermal performance curves (TPCs) typically describe an asymmetric, unimodal relationship between temperature and fitness-related traits, with a gradual rise from a lower thermal limit to an optimum, followed by a steep decline at supra-optimal temperatures (13, 14). For ectothermic arthropods, these TPCs are fundamental to understanding species distributions and predicting responses to climate change. Previous studies have documented the influence of temperature on *D. farinae* development (6, 15), but comprehensive life table analyses across a broad temperature gradient remain scarce, particularly regarding upper thermal limits that will become increasingly relevant under ongoing climate change. In addition, humidity is a critical abiotic factor for house dust mites, which absorb water vapor from the air through their cuticle; below 60% RH, most HDM species cannot maintain positive water balance (6).

Global climate change projections indicate a 1.5–4.5 °C rise in average global temperatures by the end of the 21st century (16). This warming may substantially alter the geographic distribution and population dynamics of house dust mites, with consequent impacts on human allergen exposure. Understanding the thermal biology of *D. farinae* is therefore critical for (1) defining thermal optima and limits for development and reproduction, (2) constructing predictive population models, and (3) assessing future allergen exposure risks under climate change scenarios.

Compared with previous study, which provided earlier baseline observations on *D. farinae* under more limited experimental conditions (15), the present study extends the literature by combining a broader thermal gradient with age-stage, two-sex life table analysis, thereby resolving stage-specific survival, fecundity, and population-growth responses and defining both lower and upper thermal limits. The results supported all three hypotheses: development accelerated with temperature up to an optimum and then declined at 35 °C; survival and fecundity showed unimodal thermal responses; and the life table parameters delineated a narrow optimal thermal window with clear upper limits. In this study, we systematically investigated the effects of five constant temperatures (15, 20, 25, 30, and 35 °C) on the development, survival, fecundity, and life table parameters of *D. farinae* under controlled laboratory conditions. We tested three hypotheses, (1) developmental rates increase with temperature up to an optimum, then decline at supra-optimal temperatures; (2) survival and fecundity exhibit similar unimodal thermal response patterns; and (3) life table parameters can delineate the fundamental thermal niche of this medically important species. All three tested hypotheses were supported by our results.

## Materials and methods

### Mite culture

Specimens of *D. farinae* were originally isolated from house dust samples collected from residential buildings in Zhangzhou, Fujian Province, China (24.5°N, 117.6°E). Species identity was confirmed via morphological examination under a stereomicroscope based on established taxonomic criteria. Specifically, the shape and position of the spermatheca in females and the characteristics of the aedeagus in males were used as reliable diagnostic characters that clearly distinguish *D. farinae* from *D. pteronyssinus (*17). Additionally, our stock cultures have been maintained under laboratory conditions for over three years with consistent morphological verification at each subculture. Stock cultures were maintained in glass rearing chambers (10 cm diameter × 5 cm height) with a 1:1:1 (w/w) mixture of yeast powder, wheat germ, and dried porcine liver as a food source. Cultures were reared at 25 ± 1 °C and 75 ± 5% relative humidity (RH) under continuous darkness for a minimum of three generations prior to use in experiments.

### Experimental design

Experiments were conducted in programmable environmental chambers (PRX-250B, Saifu, China) set to five constant temperature regimes: 15, 20, 25, 30, and 35 °C (± 0.5 °C). Relative humidity was maintained at 75 ± 5% using saturated sodium chloride solutions, following the method of Winston and Bates (18). We fixed humidity at this moderate level to isolate the effects of temperature as the sole experimental variable, acknowledging that temperature-humidity interactions are ecologically important and warrant separate investigation. All experiments were performed under continuous darkness.

### Development and survival of immature stages

Gravid female mites were randomly selected from stock cultures and transferred individually to 1 mL microcentrifuge tubes containing rearing medium. After a 24-hour oviposition period, females were removed, and eggs were monitored daily under a stereomicroscope (SZX7, Olympus, Japan). For each temperature treatment, 60 eggs were individually tracked; 45 eggs were used for the 35 °C treatment due to expected high mortality. The reduced sample size at 35 °C (45 eggs vs. 60 eggs at other temperatures) was based on preliminary trials showing >50% pre-adult mortality at this temperature. This adjustment ensured that sufficient adults would be available for fecundity assays while maintaining adequate statistical power for survival and development analyses. Bootstrap resampling for life table parameters accounts for treatment-specific sample sizes.

Developmental progress was recorded daily at a consistent time for each life stage: egg, larva, protonymph, tritonymph, and adult. Stage transitions were verified by the presence of exuviae. Mortality was recorded daily for each stage. Individuals that died before completing development were excluded from developmental duration calculations but included in survival rate estimates.

### Adult longevity and fecundity

Newly emerged adult females and males (< 24 hours post-eclosion) were paired (one female + one male) and placed individually in rearing tubes with fresh medium. Thirty pairs were established for each temperature treatment, with 18 pairs for the 35 °C treatment. Tubes were checked daily to record pre-oviposition period (time from adult emergence to first oviposition), oviposition period, post-oviposition period, daily fecundity, and adult longevity. Males that died were replaced to ensure continuous mating opportunity for females.

### Life table analysis

Life table parameters were calculated according to age-stage, two-sex life table theory (19, 20) using the TWOSEX-MSChart program (21). The following population parameters were estimated as below.

Age-stage specific survival rate (*s_xj_*): the probability that a newly laid egg survives to age *x* and stage *j*

Age-specific survival rate (*l_x_*): the probability that a newly laid egg survives to age *x*

Age-specific fecundity (*m_x_*): the mean number of female offspring produced per female at age *x*

Net reproductive rate (*R_0_*): Σ*l_x_m_x_*

Intrinsic rate of increase (*r_m_*): calculated via the Euler-Lotka equation: Σe^(−*r*(*x* + 1))*l_x_m_x_* = 1

Finite rate of increase (*λ*): e^(*r_m_*)

Mean generation time (*T*): ln(*R_0_*)/*r_m_*

Doubling time (DT): ln(2)/*r_m_*

Standard errors (SE) and 95% confidence intervals for all life table parameters (r_m_, R_0_, λ, T, DT) were estimated using the bootstrap procedure with 100,000 resamples implemented in TWOSEX-MSChart. Pairwise comparisons between temperature treatments were performed using the paired bootstrap test, which accounts for the non-normal distribution of life table parameters (22). Differences were considered statistically significant at P< 0.05.

### Statistical analysis

One-way analysis of variance (ANOVA) was used to compare developmental duration, adult longevity, and fecundity across temperature treatments. Means were separated using Tukey’s honestly significant difference (HSD) test at a significance level of *P* < 0.05. Percentage data (survival rates) were arcsine square-root transformed prior to analysis to meet ANOVA assumptions of normality and homogeneity of variance. All statistical analyses were performed using SPSS 26.0 (IBM Corp., Armonk, NY, USA). For life table parameters, bootstrap-derived standard errors and paired bootstrap tests were used as the primary method for statistical comparison among temperatures, consistent with standard practice in age-stage, two-sex life table studies.

Additionally, development rate (1/days) was fitted to the Brière-1 model (23).


$$\text{r}\left(\text{T}\right)=\text{aT}\left(\text{T}-{\text{T}}_{0}\right)\sqrt{\text{T\_L}-\text{T}}$$

where T is temperature (°C), T_0_ is the lower developmental threshold, T_L is the upper thermal limit, and a is an empirical constant. This nonlinear model provides biologically interpretable parameters for thermal sensitivity and is reported in the supplementary materials (**Supplementary Table 1**).

## Results

### Developmental duration

**Figure 1:**
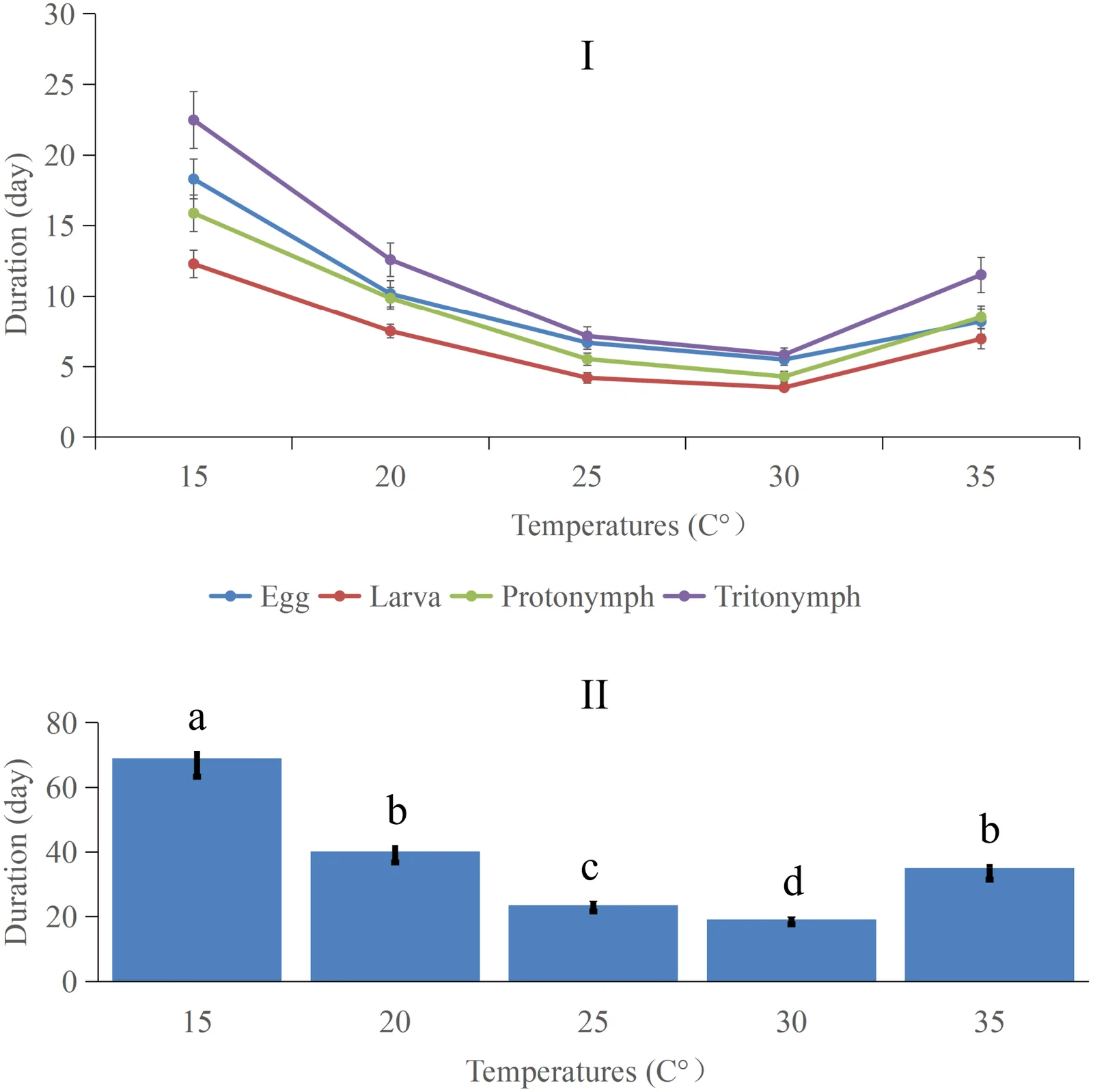
Effects of temperature on developmental duration of Dermatophagoides farinae. (I) Developmental duration of four immature stages (egg, larva, protonymph, and tritonymph) across five constant temperatures. Data points represent means with standard error (SE) bars. (II) Total pre-adult developmental duration at five constant temperatures. Bars represent means with SE; different lowercase letters above bars indicate significant differences among temperatures (one-way ANOVA followed by Tukey’s HSD test, P< 0.05).

Temperature significantly affected the developmental duration of all pre-adult stages of *D. farinae* (**Figure 1**). Across all four immature stages, developmental time decreased progressively as temperature increased from 15 to 30 °C, but increased markedly at 35 °C, indicating strong thermal inhibition at the highest temperature. As shown in **Figure 1** I, the tritonymph stage had the longest duration at all temperatures, ranging from 5.84 days at 30 °C to 22.48 days at 15 °C, followed by the protonymph, egg, and larval stages. Each stage exhibited a consistent U-shaped thermal response pattern, with the shortest duration consistently occurring at 30 °C. Total pre-adult development time (**Figure 1** II) differed significantly among temperature treatments (one-way ANOVA, P< 0.05). The shortest total development was recorded at 30 °C (19.14 days), which was 3.6-fold faster than at 15 °C (68.98 days). At 35 °C, total development time increased to 35.18 days clearly demonstrating the deleterious effects of supra-optimal temperatures on *D. farinae* development. Additionally, the pre-adult developmental rate (1/days) was fitted to the Brière-1 nonlinear model. The estimated thermal parameters were as follows: lower developmental threshold (*T*_0_) = 9.9 °C, optimal temperature for development (*T*_opt_) = 30.2 °C, upper thermal limit (*T*_max_) = 35.9 °C, and empirical constant *a* = 3.338 × 10^−4^ day^−1^ °^C−2^, with a model goodness-of-fit *R*^2^ = 0.987. The fitted curve and model equation are provided in **Supplementary Table 1** (Supporting Information).

### Stage-specific survival

Survival rates varied significantly across temperatures and developmental stages (**Table 1**). The highest survival rates were observed at 25 °C for all stages, followed closely by 30 °C. Survival declined markedly at both temperature extremes.

**Table 1:** Stage-specific survival rates (%) of *Dermatophagoides farinae* at five constant temperatures.

Stage	15 °C	20 °C	25 °C	30 °C	35 °C
Egg	72.5 ± 3.2 a	85.2 ± 2.8 b	94.8 ± 1.7 c	92.3 ± 2.1 c	65.8 ± 3.5 d
Larva	68.3 ± 3.5 a	82.5 ± 3.1 b	92.5 ± 2.0 c	90.8 ± 2.3 c	58.2 ± 3.7 d
Protonymph	75.2 ± 3.0 a	88.6 ± 2.4 b	96.2 ± 1.4 c	93.5 ± 1.9 bc	62.5 ± 3.3 d
Tritonymph	70.8 ± 3.4 a	85.3 ± 2.7 b	94.5 ± 1.6 c	91.2 ± 2.2 bc	55.3 ± 3.9 d
Egg to adult	52.3 ± 3.8 a	75.8 ± 2.9 b	88.6 ± 2.1 c	82.4 ± 2.6 bc	35.7 ± 4.2 d

Means within a row followed by different lowercase letters are significantly different (Tukey’s HSD on arcsine-transformed data, P< 0.05). ANOVA statistics: Egg-to-adult survival (F_4,25_ = 89.6, P< 0.001).

Egg-to-adult survival reached 88.6% at 25 °C, indicating near-optimal developmental conditions. At 35 °C, only 35.7% of eggs successfully developed to adults, with particularly high mortality during the tritonymph stage (55.3% survival). At 15 °C, 52.3% of eggs completed development, with prolonged developmental time contributing to increased cumulative mortality. Egg-to-adult survival represents the cumulative probability that an egg successfully completes all developmental stages (egg, larva, protonymph, tritonymph) and emerges as an adult. It was calculated as the product of stage-specific survival rates across all pre-adult stages, which equals the proportion of the initial 60 (or 45) eggs that reached adulthood.

### Adult longevity and reproduction

Temperature significantly affected adult longevity and all reproductive parameters (**Table 2**). Female longevity was greatest at 25 °C (125.4 days) and shortest at 35 °C (45.2 days). Male longevity followed a similar pattern but was consistently shorter than female longevity across all temperatures.

**Table 2:** Adult longevity and reproductive parameters of *Dermatophagoides farinae* at five constant temperatures.

Parameter	15 °C	20 °C	25 °C	30 °C	35 °C
Female longevity (days)	85.6 ± 7.8 a	105.8 ± 9.2 b	125.4 ± 10.5 c	95.8 ± 8.4 d	45.2 ± 4.6 e
Male longevity (days)	45.2 ± 5.1 a	58.6 ± 6.3 b	72.5 ± 7.4 c	55.3 ± 5.7 b	28.5 ± 3.2 d
Pre-oviposition period (days)	8.5 ± 1.2 a	5.2 ± 0.8 b	3.2 ± 0.5 c	2.8 ± 0.4 c	6.5 ± 0.9 d
Oviposition period (days)	52.3 ± 6.4 a	72.5 ± 7.8 b	85.6 ± 9.1 c	65.2 ± 7.0 d	25.8 ± 3.5 e
Total fecundity (eggs/female)	38.5 ± 4.2 a	62.8 ± 5.7 b	85.2 ± 7.1 c	72.5 ± 6.3 d	22.3 ± 2.8 e
Daily fecundity (eggs/day)	0.74 ± 0.09 a	0.87 ± 0.10 ab	1.00 ± 0.11 b	1.11 ± 0.12 c	0.86 ± 0.10 ab

Means within a row followed by different lowercase letters are significantly different (Tukey’s HSD, P< 0.05). All parameters showed significant temperature effects (ANOVA, all P< 0.001).

Total fecundity peaked at 25 °C with an average of 85.2 eggs per female, while daily oviposition rate was highest at 30 °C (1.11 eggs/day). At 35 °C, both total and daily fecundity were severely reduced, with females producing only 22.3 eggs on average. The pre-oviposition period was shortest at 30 °C (2.8 days) and longest at 15 °C (8.5 days).

### Life table parameters

Population growth parameters derived from life table analysis showed clear temperature-dependent patterns (**Table 3**). The intrinsic rate of increase (*r_m_*), a comprehensive measure of population growth potential, was highest at 30 °C (0.0598 ± 0.0021 day^−1^) and 25 °C (0.0506 ± 0.0018 day^−1^).

**Table 3:** Life table parameters of *Dermatophagoides farinae* at five constant temperatures.

Parameter	15 °C	20 °C	25 °C	30 °C	35 °C
Net reproductive rate (*R_0_*)	5.075 ± 0.412 a	16.680 ± 0.895 b	33.960 ± 1.204 c	25.906 ± 1.037 d	1.476 ± 0.215 e
Intrinsic rate of increase (*r_m_*, day^−1^)	0.0157 ± 0.0011 a	0.0345 ± 0.0015 b	0.0506 ± 0.0018 c	0.0598 ± 0.0021 d	0.0072 ± 0.0009 e
Finite rate of increase (*λ*, day^−1^)	1.0158 ± 0.0011 a	1.0351 ± 0.0015 b	1.0519 ± 0.0018 c	1.0616 ± 0.0021 d	1.0072 ± 0.0009 e
Mean generation time (*T*, days)	103.65 ± 2.14 a	81.55 ± 1.76 b	69.70 ± 1.42 c	54.40 ± 1.15 d	54.20 ± 1.31 d
Doubling time (DT, days)	44.23 ± 3.12 a	20.09 ± 0.87 b	13.70 ± 0.49 c	11.59 ± 0.41 d	96.53 ± 12.05 e
Gross reproductive rate (GRR)	19.25 ± 1.56 a	31.40 ± 1.68 b	42.60 ± 1.92 c	36.25 ± 1.75 d	11.15 ± 1.23 e

Standard errors were estimated via 100,000 bootstrap resamples. Means within a row followed by different lowercase letters are significantly different (paired bootstrap test, P< 0.05).

The net reproductive rate (*R_0_*) was maximum at 25 °C (33.96 female offspring per female), indicating that despite slightly slower development than at 30 °C, the combination of high survival and high fecundity yielded the greatest lifetime reproductive output. Mean generation time decreased from 103.65 days at 15 °C to 54.40 days at 30 °C. Population doubling time was shortest at 30 °C (11.59 days) and longest at 35 °C (96.53 days).

### Age-stage survival and fecundity curves

**Figure 2:**
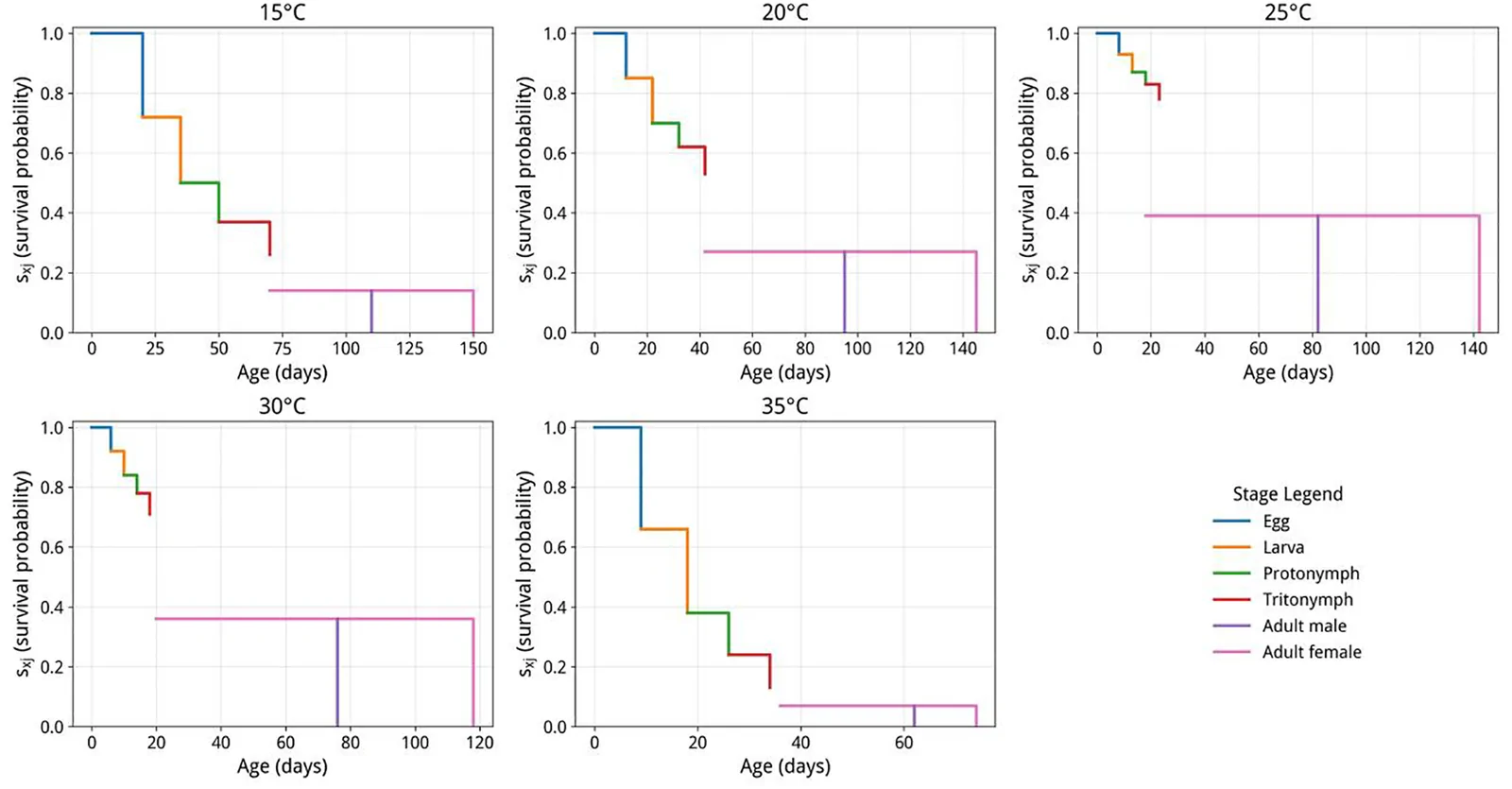
Age-stage specific survival rate (s_xj_) of Dermatophagoides farinae at five constant temperatures. Each panel shows the probability that an egg survives to age x and develops to stag.

**Figure 3:**
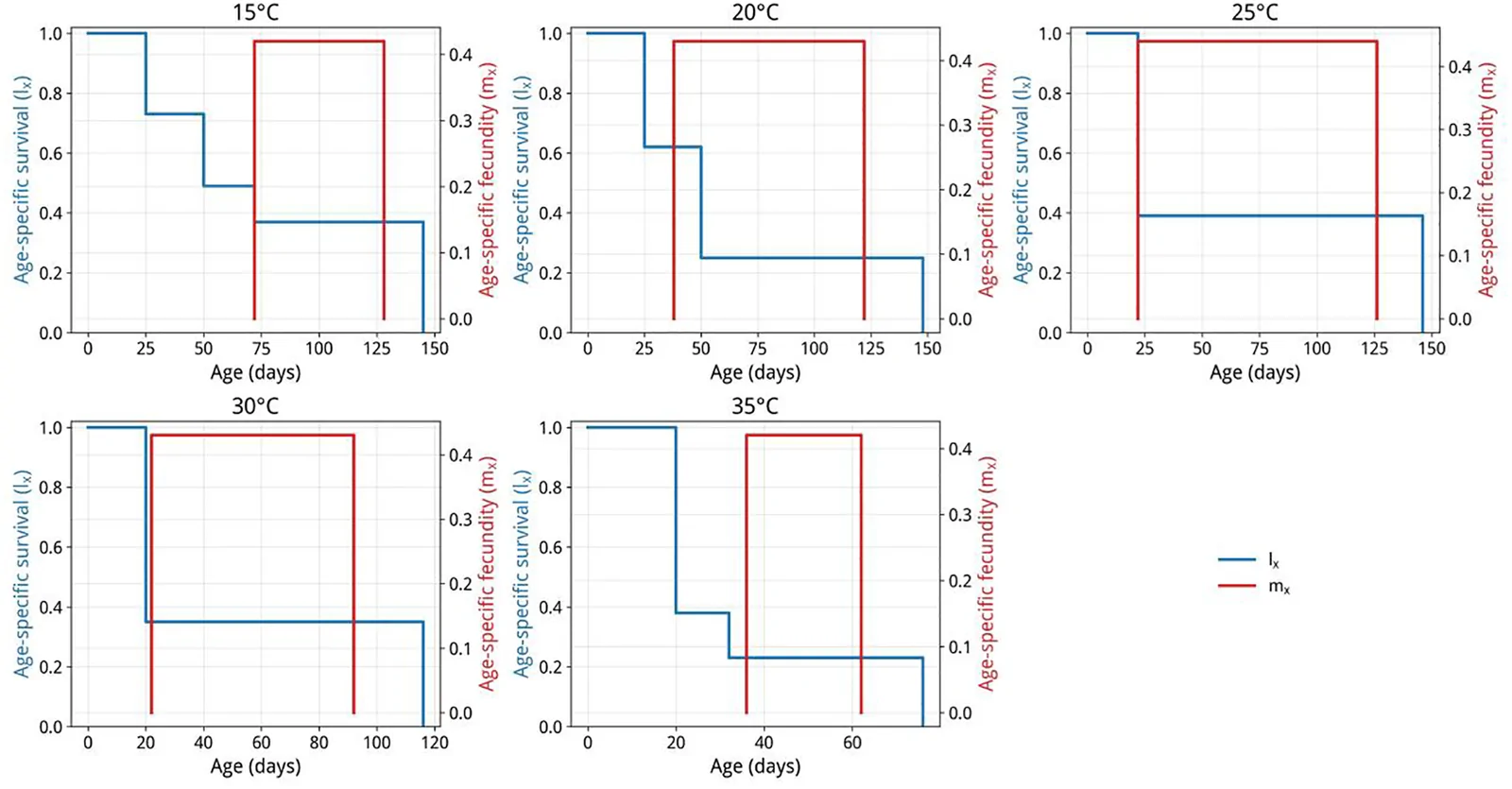
Age-specific survival rate (l_x_) and age-specific fecundity (m_x_) of Dermatophagoides farinae at five constant temperatures. Blue lines (left y-axis) represent age-specific survival; red lines (right y-axis) represent daily female offspring production per female.

**Figure 4:**
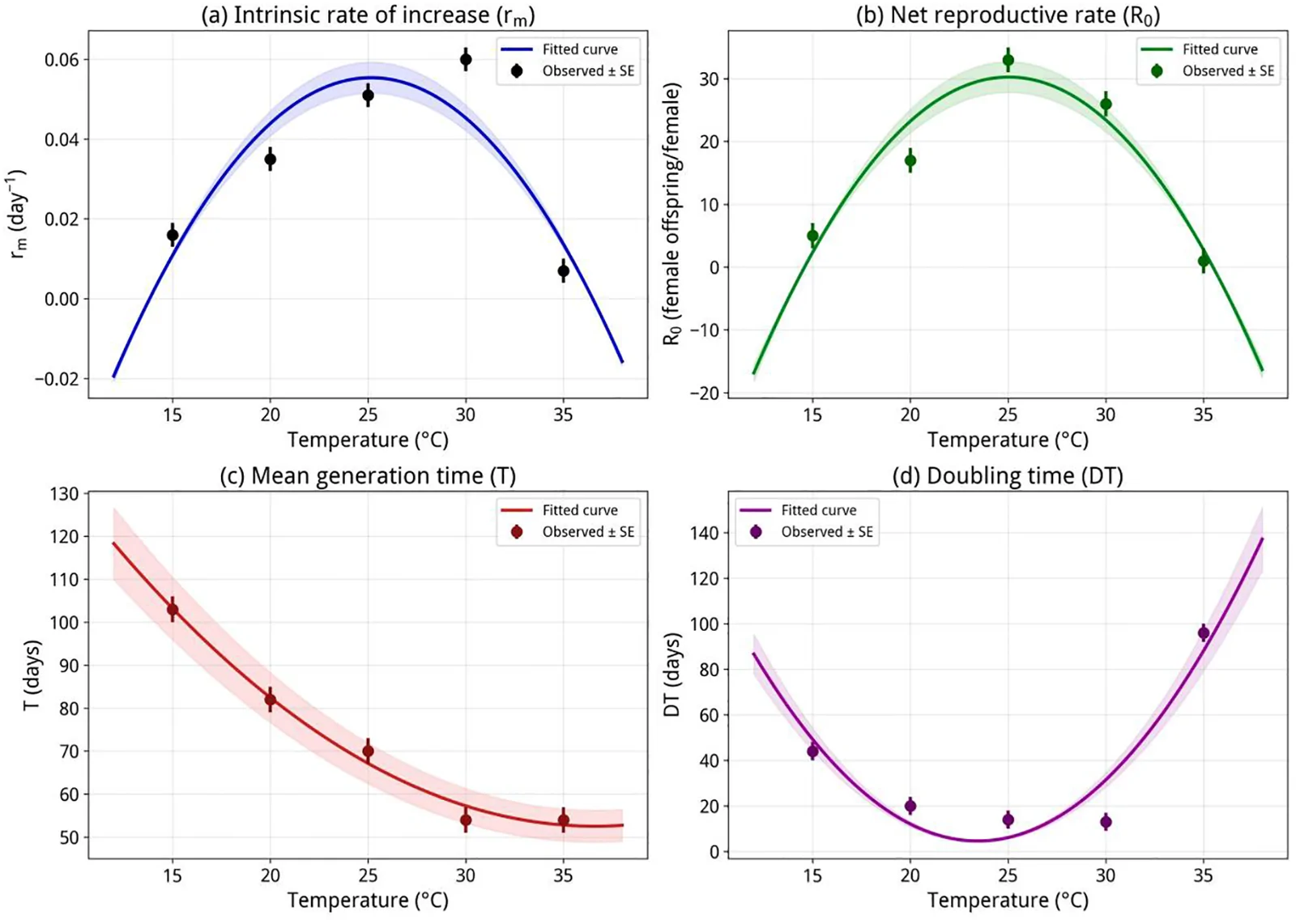
Temperature-dependent life table parameters of Dermatophagoides farinae with bootstrap-derived 95% confidence bands. **(A)** Intrinsic rate of increase (r_m_); **(B)** Net reproductive rate (R_0_); **(C)** Mean generation time (T); **(D)** Doubling time (DT). Circles represent observed values with ± SE error bars; solid lines represent quadratic fitted curves; shaded areas indicate 95% confidence intervals.

The age-stage specific survival rates (s_xj_) revealed clear stage-specific developmental trajectories at each temperature (**Figure 2**). The overlap between stages reflects variable developmental rates among individuals, a characteristic feature of the age-stage, two-sex life table approach. Age-specific survival (l_x_) and fecundity (m_x_) curves (**Figure 3**) showed that reproduction began earliest and peaked most rapidly at 30 °C, while the highest cumulative fecundity occurred at 25 °C. Temperature-dependent trends for key life table parameters with bootstrap 95% confidence bands are shown in **Figure 4**, illustrating the unimodal thermal response for r_m_ and R_0_ and the monotonic decrease in generation time with temperature up to 30 °C.

## Discussion

This study presents one of the most comprehensive thermal biology datasets for *Dermatophagoides farinae* to date, spanning a biologically relevant temperature gradient from suboptimal to supra-optimal conditions. Our results demonstrate that temperature exerts strong effects on all aspects of *D. farinae* biology, with clear optima for development, survival, and reproduction between 25 and 30 °C.

The developmental response of *D. farinae* to temperature follows the typical asymmetric thermal performance curve observed in most ectothermic organisms (24). Developmental rates accelerated in a near-linear fashion between 15 and 30 °C but declined sharply at 35 °C. The total development time of 19.14 days at 30 °C is among the shortest reported for pyroglyphid mites and is consistent with previous findings for *D. farinae (*15, 16). The asymmetric thermal performance curve observed here is consistent with thermodynamic constraints on enzyme function and metabolic rate: reaction rates increase with temperature up to an optimum, beyond which protein denaturation and physiological stress cause rapid performance decline (25). The particularly sharp decline above 30 °C suggests that *D. farinae* operates close to its thermal optimum under typical room temperatures, making it vulnerable to even moderate heat stress.

The thermal inhibition observed at 35 °C is particularly notable. At this temperature, development time increased by 84% relative to 30 °C, and survival dropped precipitously. This pattern suggests that 35 °C approaches or exceeds the upper thermal limit for *D. farinae* development. The tritonymph stage appeared most sensitive to heat stress, with only 55.3% survival at 35 °C. This stage-specific vulnerability may reflect the elevated metabolic demands associated with the final molt to adulthood, when energy requirements are highest and physiological stress has disproportionate impacts on developmental success. This hypothesis could be tested in future studies by measuring stage-specific metabolic rates or heat shock protein expression across temperatures.

The survival patterns observed in this study have important implications for understanding *D. farinae* population dynamics. The high egg-to-adult survival (>88%) at 25 °C confirms this temperature as optimal for *D. farinae*. The sharp decline in survival at 35 °C (35.7% egg-to-adult) indicates that even moderate sustained heat stress can severely limit population establishment and persistence. Fecundity showed a similar unimodal thermal response, with maximum total egg production at 25 °C (85.2 eggs per female) and maximum daily oviposition rate at 30 °C (1.11 eggs per day). This trade-off between total lifetime fecundity and daily reproductive output explains why *r_m_* peaked at 30 °C despite lower *R_0_*, faster development and earlier reproduction contribute more strongly to population growth rate than does total lifetime fecundity, a well-documented pattern in age-structured population ecology (26, 27).

The life table parameters provide critical insights into *D. farinae* population ecology. The intrinsic rate of increase (*r_m_* = 0.0598 day^−1^ at 30 °C) indicates high population growth potential under optimal conditions — populations can double in less than 12 days. This rapid growth capacity explains how mite populations can reach allergenically significant densities within weeks under favorable indoor conditions.

The substantial reduction in *r_m_* at temperature extremes (0.0157 day^−1^ at 15 °C, 0.0072 day^−1^ at 35 °C) reflects the relatively narrow thermal niche of *D. farinae*. This thermal specialization has important implications for (1) the geographic distribution of this species, which is largely restricted to temperate and subtropical regions; (2) seasonal population fluctuations, with peaks typically occurring in spring and autumn when indoor temperatures approach 25 °C; and (3) potential range shifts under climate change.

### Climate change implications

The thermal performance data generated in this study provide essential baseline parameters for future population modeling efforts aimed at assessing how climate change may affect house dust mite populations and associated allergen exposure. Our finding of a thermal optimum between 25 and 30 °C suggests that in regions where current indoor temperatures are below this range (e.g., cooler temperate zones), moderate warming could potentially increase *D. farinae* population growth rates and extend seasonal activity periods. Conversely, in tropical and subtropical regions where summer indoor temperatures already approach or exceed 35 °C, further warming could suppress mite populations. However, we emphasize that these are qualitative interpretations based on laboratory constant-temperature data from a single population. Real-world indoor environments feature diurnal temperature fluctuations, variable humidity, and microclimatic heterogeneity that cannot be captured by constant-temperature experiments. Formal degree-day models, population viability analyses, and climate projection downscaling will be required to generate quantitative predictions of future HDM distributions and allergen exposure risks.

Our findings have important implications for predicting house dust mite populations and associated allergen exposure under climate change scenarios. The observed thermal optimum of 25–30 °C suggests that moderate warming of 1–2 °C could expand the geographic range of *D. farinae* into currently cooler temperate regions where summer temperatures presently limit population growth, prolong the seasonal activity period in temperate zones to extend allergen exposure seasons, and increase population growth rates in regions currently below thermal optima. However, further warming exceeding 3 °C could eventually exceed the thermal tolerance of *D. farinae*, particularly in tropical and subtropical regions where summer temperatures already approach 35 °C, which would create a shifting pattern of allergen risk characterized by elevated exposure in temperate zones and potential reductions in the warmest regions.

Several limitations of this study should be acknowledged. First, our experiments used constant temperatures, while natural indoor environments experience diurnal temperature fluctuations. Fluctuating temperatures may produce different developmental outcomes, as brief exposure to higher or lower temperatures can modulate thermal performance (28, 29). Second, we maintained constant RH at 75%, while humidity interacts with temperature to affect mite water balance and physiological performance (5, 6, 30). Future studies should incorporate these interacting abiotic factors to improve the realism of population projections.

## Conclusions

This study demonstrates that *Dermatophagoides farinae* has a relatively narrow thermal niche, with optimal development and population growth occurring between 25 and 30 °C. Temperatures above 35 °C cause significant thermal stress, resulting in prolonged development, reduced survival, and diminished fecundity. The comprehensive life table data generated here provide essential parameters for population modeling and predicting the impacts of climate change on house dust mite distributions and allergen exposure risks. These findings can inform public health strategies for managing allergic diseases in a changing climate.

## Data Availability

The original contributions presented in the study are included in the article/**Supplementary Material**. Further inquiries can be directed to the corresponding authors.

## Supplementary material

Supplementary Table 1 Raw experimental data of Dermatophagoides farinae. This Excel file contains raw data and summary statistics for developmental duration, stage-specific survival rates, individual and summary fecundity data, complete life table parameters, and age-stage specific life table (lx, mx) data.
